# Giant primary dural lymphoma with vault involvement

**DOI:** 10.11604/pamj.2021.38.117.27046

**Published:** 2021-02-03

**Authors:** Mohamed Moutaoukil, Brahim Eljebbouri

**Affiliations:** 1 Fourth Military Hospital Dakhla, Dakhla, Morocco

**Keywords:** Giant primary dural lymphoma, frontal headaches, computed tomography

## Image in medicine

A 52-year-old immunocompetent man presented with frontal headaches. Physical examination showed a painful frontal hump (A). Computed tomography (CT) and magnetic resonance imaging (MRI) scans revealed a giant bifrontal extra-axial tumor (B, C). He benefited from total removal of the tumor. Pathological examination of the resected mass led to the diagnosis of anaplastic large B-cell lymphoma (cell immunopositivity for CD30 and anaplastic lymphoma kinase) (D). He was transferred to the oncology unit for chemo and radiotherapy with good clinical evolution. Primary dural lymphoma (PDL) is extremely rare, accounting for 7% of all primary central nervous system lymphomas. PDL usually involves sites that are rich in meningothelial cells, and results in a localized mass or plaque-like thickening of the dura that radiologically resembles a number of other diseases, such as meningioma or subdural hematomas. Although there is as yet no optimal treatment for PDL, surgical excision followed by radiotherapy and chemotherapy are recommended.

**Figure 1:**
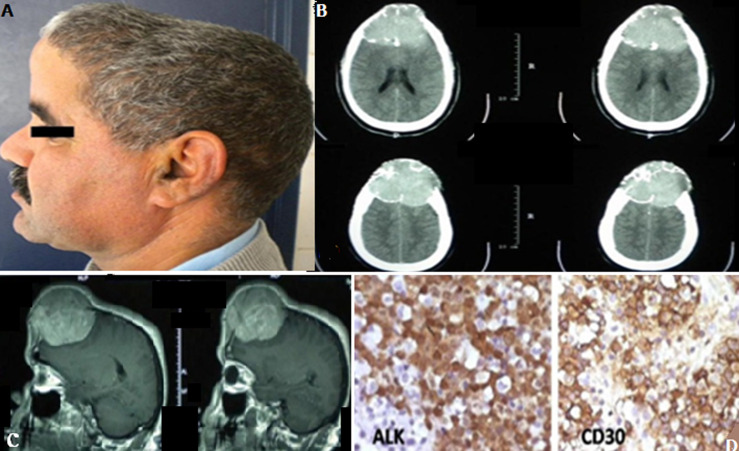
A) picture of the patient with frontal hump; B) axial CT scan after injection of contrast medium showing the appearance of erosive and invasive frontal tumor; C) magnetic resonance imaging (MRI) sagittal T1-weighted images following gadolinium administration; a heterogeneously enhancing and lobulated dural mass developed within the superior sagittal sinus (SSS), and was surrounded by localized cerebral edema; D) cell immunopositivity for CD30 and anaplastic lymphoma kinase (IHC stain ×200)

